# Robotic-assisted versus free-hand techniques in adult spinal deformity surgery: a comparative analysis of postoperative outcomes

**DOI:** 10.1007/s11701-025-02543-7

**Published:** 2025-07-11

**Authors:** Ved A. Vengsarkar, Ariaz Goudarzi, Jialun Chi, Arsany Yassa, Jesse Wang, Lawal Labaran, Xudong Li

**Affiliations:** 1 Department of Orthopaedic Surgery, Rutgers New Jersey Medical School, Newark, NJ USA; 2 Department of Orthopaedic Surgery, University of Virginia School of Medicine, 2280 Ivy Rd, Charlottesville, VA 22903 USA; 3 Department of Plastic Surgery, University of Missouri School of Medicine, Columbia, MO USA

**Keywords:** Adult spinal deformity, Robotic, Free hand, Complications, Long segment, Retrospective

## Abstract

Adult spinal deformity (ASD) represents a challenging area in spinal surgery due to its complexity and potential for postoperative complications. While robotic systems have promised enhanced precision for pedicle screw placement, improved alignment, and potentially fewer complications, the comparative efficacy and morbidity of robotic-assisted (RA) versus free-hand (FH) techniques remains underexplored in the context of ASD. This study aims to compare postoperative outcomes in patients undergoing RA techniques versus FH surgery to manage ASD. A retrospective review was performed and included patients over 18 who underwent a posterior fusion of at least 7 segments from 2015 to 2022. Two separate cohorts were created based on the use of RA or FH. Patients were matched with a 1:5 ratio based on age, sex, utilization of posterior column osteotomy, utilization of three-column osteotomy, utilization of pelvic fixation, number of instrumented levels, and prior lumbar revision. Multivariate logistic regression was used to compare 90-day complications and healthcare utilization. The RA group demonstrated a significantly lower incidence of atelectasis (3.0% vs. 6.8%; p=0.018), respiratory failure (2.7% vs. 6.7%; *p* = 0.010), pleural effusion (3.3% vs. 7.0%; *p* = 0.021), and pneumonia (2.1% vs. 6.0%; *p* = 0.004) compared to the FH group. In addition, the RA group also demonstrated a reduced incidence of spinal cord deficits (1.2% vs. 3.4%; *p* = 0.048) and a shorter average length of stay (LOS), in days (8.1 +/− 8.3 vs. 10.2 +/− 12.8; *p* = 0.009). In the present study, we discover that robotic assistance leads to improved short-term outcomes, including reduced rates of pulmonary (atelectasis, pneumonia, pleural effusion, respiratory failure) and neurologic (spinal cord deficit) complications, as well as shorter hospital stays. While RA systems have previously demonstrated technical advantages in pedicle screw placement, this study provides evidence of meaningful clinical benefits as well.

## Introduction

Adult spinal deformity (ASD) encompasses a set of disorders that cause deformities in the coronal and sagittal planes, including conditions such as scoliosis, kyphosis, and flatback [1, 2]. These disorders typically affect patients over the age of 60, with factors such as reduced bone mineral density and imbalanced gait contributing to the progression of ASD [1]. Despite the varied presentations,  ASD poses significant challenges in spinal surgery due to the complexity of deformities and the elevated risk of postoperative complications [3]. Patients who are older or have extensive comorbidities are especially vulnerable [4]. Deformities of the spine can have many adverse consequences such as pain, disability, psychosocial stress, and an overall diminished quality of life [1]. The first-line treatment is typically non-operative with bracing, physical therapy, and pain management [1, 5]. However, debilitating symptoms often necessitate surgical correction, ranging from limited decompression to long-segment fusions [6, 7]. These surgeries carry considerable risk, including hardware misplacement, neural injuries, and infection [8, 9].

For decades, the gold standard of ASD surgery was free-hand surgical techniques [10, 11]. These traditional methods involve manually placing pedicle screws without real-time intraoperative guidance and relying heavily on the experience of the surgeon and their anatomical knowledge. This technique is widely used due to its reliability and safety, with one study finding a 96% accuracy rate for pedicle screw placement [12]. Another study, by Kim et al., found no significant neurological, vascular, or visceral complications over a 10-year follow-up period [13].

The free-hand method has been effective in many cases in the past; however, it does not preclude the technique from having limitations when compared to robotic-assisted surgery. These robotic systems help enhance the precision of the pedicle screw placement by providing real-time intraoperative feedback guided by preoperative plans, allowing surgeons to plan precise screw trajectories using 3D spinal models [14]. Previous studies have shown that robotic-assisted surgery increases the accuracy of screw trajectories, minimizes radiation exposure, and decreases postoperative stays when compared to free-hand techniques [15–17]. There is still debate ; however, many studies suggest that robotic-assisted surgeries may take longer [15]. Despite the breadth of research into the comparative efficacy of robotic-assisted versus free-hand techniques, the field remains underexplored in the context of ASD. With ASD being incredibly complex, every advantage can be beneficial; consequently, investigating the free-hand method vs robotic-assisted is of paramount importance.

Thus, this study aims to retrospectively analyze patients who underwent ASD correction with either robotic-assisted or free-hand methods and compare immediate postoperative outcomes between these groups including complication rates, length of stay (LOS), and 90-day readmissions. This study aims to inform clinical practice and guide surgical decision-making for ASD correction surgery.

## Methods

### Data source

The data used in this analysis were obtained from the multiplayer database PearlDiver (PearlDiver, Inc., Colorado Springs, Colorado, USA), which contains records for over 161 million patients. These data included procedural and demographic information encoded with International Classification of Diseases (ICD) and Current Procedural Terminology (CPT) codes. In full compliance with the Health Insurance Portability and Accountability Act, all data are de-identified.

### Study population

Patients included in this study were identified from CPT codes defining posterior fusion of at least 7 segments (CPT-22802, CPT-22843, CPT-22804, CPT-22844) from 2015 to 2022. Patients with prior history of malignancy or metastasis, trauma, and infection, or age less than 18 years were excluded. Two separate cohorts were created based on the use of robotic assistance (CPT-S2900, ICD-10-P-8E0W0CZ, ICD-10-P-8E0W3CZ, ICD-10-P-8E0W4CZ, ICD-10-P-8E0W7CZ, ICD-10-P-8E0W8CZ, ICD-10-P-8E0WXCZ). Utilization of three-column osteotomy, posterior column osteotomy, and pelvic fixation in both groups was also determined. All patients had a minimum of 2-year follow-up. Patients were matched with a 1:5 ratio based on age, sex, CCI, utilization of posterior column osteotomy, utilization of three-column osteotomy, utilization of pelvic fixation, number of instrumented levels, and prior lumbar revision.

### Outcomes

Patients in both groups were evaluated for 90-day medical complications: arrhythmia, myocardial infarction, cardiac arrest, atelectasis, respiratory failure, pleural effusion, pulmonary embolism, deep vein thrombosis, cerebrovascular accident, delirium, ileus, bowel obstruction, electrolyte imbalance, renal failure, urinary retention, pneumonia, sepsis, and UTI. 90-day surgical complications include dural tear, nerve root injury, vascular injury, visceral injury, transfusion, nerve root motor deficit, nerve root sensory deficit, radiculopathy, spinal cord deficit, cauda equina deficit, wound dehiscence, seroma, hematoma, deep wound infection, superficial wound infection, painful or prominent instrumentation, instrumentation failure, proximal junctional failure, and reoperation. Length of stay, 90-day emergency department visits, and 90-day inpatient readmissions were assessed.

## Results

Upon applying exclusion criteria and matching, 329 patients who underwent robotic-assisted (RA) ASD correction were matched with 1623 patients who underwent free-hand (FH) ASD correction. The mean age of patients in the RA group was 62.3 ± 17.2 and 62.2 ± 17.0 in the FH group, showing no significant difference (*p* = 0.966). Gender distribution did not significantly differ between the two groups, with females constituting 50.8% in both groups (*p* = 1.000). Comorbidities were assessed using the CCI and both groups had a mean score of 2.9±2.5 (*p* = 0.819). Other significant chronic comorbid conditions that were significantly different include higher BMI 30–40 (21.3% vs. 15.0%; *p* = 0.005), smoking use (32.2% vs. 26.3%; *p* = 0.033), and depression (45.0% vs. 36.1%; *p* = 0.002) in the RA group compared to the FH group (Table 1). Comparing surgical details, a staged anterior surgery was more common in the RA group compared to the FH group (7.9% vs. 4.1%; *p* = 0.005).

**Table 1:** Demographic, comorbidities, and surgical parameters for 1952 patients undergoing adult spinal deformity correction stratified by use of robotic technologies

		RA *n* = 329		FH *n* = 1623		*p*-value
*Demographics*						
Age (mean±SD)		62.3 ± 17.2		62.2 ± 17.0		0.966
Sex (female)		167	50.8%	825	50.8%	1.000
Region	Midwest	30	9.1%	372	22.9%	< 0.001*
	Northeast	133	40.4%	276	17.0%	
	South	114	34.7%	655	40.4%	
	West	52	15.8%	320	19.7%	
*Comorbidities*						
CCI (mean±SD)		2.9 ± 2.5		2.9 ± 2.5		0.819
BMI 30–40		70	21.3%	243	15.0%	0.005*
BMI 40+		25	7.6%	112	6.9%	0.738
Smoke		106	32.2%	427	26.3%	0.033*
Diabetes mellitus		114	34.7%	637	39.2%	0.133
Chronic kidney disease		58	17.6%	274	16.9%	0.803
Depression		148	45.0%	586	36.1%	0.002*
Osteoporosis		15	4.6%	77	4.7%	0.884
Surgical details						
No. of instrumented levels	≤ 11	305	92.7%	1507	92.9%	1.000
	> 11	29	8.8%	138	8.5%	0.939
Sacropelvic fixation		264	80.2%	1297	79.9%	0.892
Staged anterior surgery		26	7.9%	67	4.1%	0.005*
Previous fusion		77	23.4%	380	23.4%	1.000

*RA* Robotic-assisted, *FH* Free hand, *CCI* Charlson comorbidity index, *BMI* Body mass index

Boldface indicates statistical significance with *p* < 0.05

Regarding pulmonary complications, a lower incidence of atelectasis (3.0% vs. 6.8%; *p* = 0.018), respiratory failure (2.7% vs. 6.7%; *p* = 0.010), pleural effusion (3.3% vs. 7.0%), and pneumonia (2.1% vs. 6.0%; *p* = 0.004) within the RA group was observed compared to the FH group (Table 2). Comparing surgical complications, there was a significantly lower rate of spinal cord deficit in the RA group compared to the FH group (1.2% vs. 3.4%; *p* = 0.048). The average length of stay (LOS) was significantly lower in the RA group compared to the FH group as well (8.1 ± 8.3 vs. 10.2 ± 12.8; *p* = 0.009) (Table 2).

**Table 2:** Multivariate analysis of rates of 90-day medical and surgical complications for both groups

	RA *n* = 329		FH *n* = 1623		Adjusted OR (95% CI)	*p*-value
*Medical*						
Arrhythmia	38	11.6%	205	12.6%	0.93 (0.63–1.35)	0.733
Myocardial infarction	8	2.4%	46	2.8%	0.86 (0.37–1.76)	0.707
Cardiac arrest	1	0.3%	10	0.6%	0.64 (0.03–3.55)	0.683
Atelectasis	10	3.0%	110	6.8%	0.45 (0.21–0.83)	0.018*
Respiratory failure	9	2.7%	108	6.7%	0.40 (0.18–0.76)	0.010*
Pleural effusion	11	3.3%	114	7.0%	0.47 (0.23–0.85)	0.021*
PE	0	0.0%	16	1.0%	~	~
DVT	5	1.5%	43	2.6%	0.49 (0.16–1.15)	0.144
CVA	4	1.2%	26	1.6%	0.73 (0.21–1.93)	0.572
Delirium	0	0.0%	22	1.4%	~	~
Ileus	4	1.2%	46	2.8%	0.40 (0.12–1.01)	0.088
Bowel obstruction	2	0.6%	18	1.1%	0.52 (0.08–1.84)	0.388
Electrolyte imbalance	17	5.2%	98	6.0%	0.86 (0.48–1.43)	0.584
Renal failure	19	5.8%	107	6.6%	0.83 (0.48–1.37)	0.500
Urinary retention	13	4.0%	106	6.5%	0.57 (0.30–0.99)	0.064
Pneumonia	7	2.1%	98	6.0%	0.31 (0.13–0.64)	0.004*
Sepsis	8	2.4%	38	2.3%	1.04 (0.44–2.17)	0.920
UTI	22	6.7%	157	9.7%	0.69 (0.42–1.08)	0.127
*Surgical*						
Dural tear	19	5.8%	130	8.0%	0.70 (0.43–1.15)	0.165
Nerve root injury	6	1.8%	42	2.6%	0.69 (0.29–1.65)	0.416
Vascular injury	1	0.3%	8	0.5%	0.61 (0.07–4.93)	0.648
Visceral injury	0	0.0%	4	0.2%	~	~
Transfusion	12	3.6%	69	4.3%	0.92 (0.46–1.67)	0.796
Nerve root motor deficit	12	3.6%	66	4.1%	0.86 (0.43–1.57)	0.662
Nerve root sensory deficit	2	0.6%	18	1.1%	0.58 (0.09–2.06)	0.477
Radiculopathy	4	1.2%	20	1.2%	0.97 (0.27–2.61)	0.961
Spinal cord deficit	4	1.2%	55	3.4%	0.35 (0.10–0.87)	0.048*
Cauda equina deficit	2	0.6%	11	0.7%	0.95 (0.14-3.69)	0.954
Wound dehiscence	24	7.3%	106	6.5%	1.10 (0.67–1.73)	0.669
Seroma	9	2.7%	36	2.2%	1.50 (0.68–3.02)	0.276
Hematoma	2	0.6%	14	0.9%	1.21 (0.59–2.59)	0.569
Deep wound infection	26	7.9%	114	7.0%	1.14 (0.71–1.76)	0.564
Superficial wound infection	9	2.7%	69	4.3%	0.63 (0.31–1.28)	0.204
Instrumentation painful/prominent	0	0.0%	5	0.3%	~	~
Instrumentation failure	5	1.5%	23	1.4%	1.10 (0.36–2.73)	0.849
PJF	6	1.8%	25	1.5%	1.11 (0.44–2.72)	0.836
Reoperation	12	3.6%	80	4.9%	0.70 (0.35–1.26)	0.265
LOS (mean±SD)	8.1 ± 8.3		10.2 ± 12.8		–	0.009*
ED visit	45	13.7%	246	15.2%	0.88 (0.62–1.24)	0.495
Inpatient readmission	68	20.7%	345	21.3%	0.96 (0.72–1.29)	0.811

*PE* Pulmonary embolism, *DVT* Deep vein thrombosis, *CVA* Cerebrovascular accident, *UTI* Urinary tract infection, *SSI* Surgical site infection, *PJF* Proximal junctional failure, *LOS* Length of stay, *ED* Emergency department

Boldface indicates statistical significance with *p* < 0.05.

## Discussion

This study aimed to compare the immediate postoperative outcomes of free-hand and robotic-assisted techniques for ASD correction surgery using a retrospective analysis of a large patient database. Our findings indicate that robotic-assisted surgery is associated with improved immediate postoperative outcomes compared to the free-hand technique in the correction of ASD. In particular, there are significantly lower rates of respiratory complications, such as atelectasis and pneumonia, as well as reduced incidences of spinal deficits in patients who underwent robotic-assisted surgeries. Furthermore, the robotic-assisted surgery group had a shorter length of stay, suggesting faster recovery and fewer complications [18].

For extensive surgeries, including spinal surgeries, postoperative respiratory failure is a common issue particularly in older patients or those with comorbidities [19, 20]. The decreased incidence of these complications during robotic-assisted surgeries can possibly be attributed to reduced soft tissue manipulation or increased precision. In order to increase the accuracy of pedicle screw placement during the FH approach, surgeons have to dissect the lamina, pars, and transverse process clearly, which involves soft tissue and bone periosteum damage [21]. Generally, robotic-assisted surgeries often involve less manipulation of the surrounding tissue and thus have less soft tissue damage, especially if utilizing a minimally invasive percutaneous pedicle screw placement [22]. This decreased soft tissue damage can reduce the body’s stress response and potentially lower the risk of postoperative respiratory issues. Increased precision can lead to this decrease in surrounding tissue damage. Spinal deformities can significantly impact pulmonary function and reduce forced vital capacity (FVC) and total lung capacity (TLC) [22].

One of the most significant findings from this study was the reduction in spinal cord deficits in the robotic-assisted group. Spinal cord deficits include motor weakness, bladder dysfunction, alteration and sensation, and possible balance changes. These complications can decrease quality of life. Therefore, maintaining the spinal cord is of utmost importance in ASD correction [23, 24]. A major risk factor for temporary or permanent injury to the spinal cord in spinal surgeries is pedicle screw misplacement [25–27]. Robotic-assisted surgery, with real-time imaging and guidance, allows for more accurate screw trajectories, reducing the risk of iatrogenic injury [15]. Therefore, our results, which are in line with previous studies, demonstrate that the robotic systems’ improved accuracy correlates with a lower rate of neurological problems postoperatively [28].

The shorter length of stay (LOS) observed in the robotic-assisted group further supports the advantages of this technique and can possibly be attributed to less invasive approaches, decreased complications postoperatively, and faster recovery. Reduced LOS is often a marker of fewer complications and faster recovery, which can translate to decreased healthcare costs and improved patient satisfaction. Robotic-assisted techniques have frequently been associated with lower estimated blood loss and decreased soft tissue damage [22, 29]. This can result in faster healing and fewer post-surgical complications. However, we acknowledge that operative time, an important variable linked to postoperative complications and LOS, was not available in our dataset and represents a limitation of the study [30]. There can be a wide variety of reasons for a shorter LOS; however, the implication of decreased complications leading to the shortened LOS supports the use of robotic-assisted surgery. Future studies should consider collecting operative time data to more precisely evaluate its impact on pulmonary outcomes and length of stay.

Although our study found supporting evidence for robotic-assisted surgery, readmission and reoperation were not significantly different between the two groups. The existing literature remains conflicted regarding surgical revisions. One retrospective review and meta-analysis evaluating robot-guided lumbar fusion reported higher revision rates in free-hand technique relative to robot-assisted [31]. In contrast, a subsequent meta-analysis evaluating thoracolumbar fusion surgery suggested that postoperative revisions were not significantly lower in the robotic-guided group based on sensitivity analyses [32]. A PearlDiver study reported a higher rate of 30-day revision and readmissions in patients who underwent robotic-assisted posterior lumbar spinal fusion compared to conventional fusion techniques [33]. These findings highlight that the clinical superiority of robotic-assisted surgery remains inconclusive in spine surgery broadly, with even less clarity regarding its impact in patients with ASD and long-segment constructs.

Despite our study having promising findings, several limitations should be acknowledged. As a retrospective review, our study is subject to selection bias as the decision of whether to use robotic-assisted or free-handed techniques can be based on surgeon preference or institutional factors that may not have been randomly assigned. While we also use matching to account for differences in baseline characteristics such as age, sex, and comorbidities, there are still many unmeasured confounders that could influence results. Furthermore, our study only evaluated immediate postoperative outcomes within 90 days of surgery, which limits our ability to extrapolate long-term benefits between the two methods, including sustained functional recovery or quality of life improvements. In addition, with limitations, our study uses PearlDiver, a private analytic database, which primarily includes patients who were not randomly sampled, 65 and older, and generally from the Southern United States [34, 35]. PearlDiver also uses a unique coding interface, which brings in the potential for coding errors. Another significant limitation is a lack of radiographic data. Radiographic assessments are crucial for understanding the extent of deformity correction achieved by each surgical method, including parameters such as sagittal vertical axis (SVA), pelvic tilt, and lumbar lordosis. Without these metrics, it is challenging to evaluate whether the reduced complications and shorter hospital stays observed in the robotic-assisted group are attributable to superior alignment or other procedural factors. Our study’s robotic-assisted cohort included open and minimally invasive techniques. This heterogeneity could confound the results, as the benefits of robotic-assisted surgery may be attributed not only to the robotic guidance but also to the inherent advantages of minimally invasive approaches, such as reduced soft tissue damage, faster recovery, and decreased LOS. Ideally, future research should stratify by approach type or adjust for it in multivariate models to isolate the effects of robotics from those of minimally invasive techniques. Furthermore, our study does not differentiate between fluoroscopy-based and intraoperative CT-based registration methods. Previous research by Stewart et al. has demonstrated superior outcomes with CT-based registration, which may skew the results of this study [36]. It is also important to consider that surgeon experience and institutional variation may have influenced outcomes which could not be controlled in our study [37]. The absence of baseline coronal deformity data prevents us from stratifying patients based on the severity of their deformities, which might influence the choice of surgical technique and postoperative outcomes. The higher rate of staged anterior surgeries in the robotic-assisted group may indicate a tendency to use this approach for more complex cases, but without radiographic data, this remains speculative. Radiographic imaging would also enable a more nuanced understanding of whether robotic systems offer distinct advantages in achieving anatomical correction in severe deformities compared to free-hand methods. Future research should prioritize integrating radiographic and patient-reported outcomes to establish a clearer link between surgical precision, deformity correction, and long-term patient benefits.

## Conclusion

In conclusion, this study provides evidence that robotic-assisted surgery offers significant advantages over the free-hand technique for ASD correction, particularly in reducing respiratory and neurological complications as well as shortening hospital stays. These findings underscore the potential of robotic systems to improve patient outcomes in complex spinal deformity cases.

## Data Availability

The data utilized in this study were obtained from the PearlDiver database, a commercially available database of de-identified patient records. Access to the data is subject to a license agreement. Interested researchers may obtain access to the database through PearlDiver Inc. (www.pearldiverinc.com).
